# Sex differences in cardiorespiratory control under hypoxia: the roles of oxygen desaturation and hypoxic exposure time

**DOI:** 10.3389/fcvm.2025.1473910

**Published:** 2025-01-31

**Authors:** André Luiz Musmanno Branco Oliveira, Gabriel Dias Rodrigues, Bruno Moreira Silva, Philippe de Azeredo Rohan, Pedro Paulo da Silva Soares

**Affiliations:** ^1^ Laboratory of Experimental and Applied Exercise Physiology, Biomedical Institute, Department of Physiology and Pharmacology, Fluminense Federal University (UFF), Niterói, Brazil; ^2^ Department of Clinical Sciences and Community Health, University of Milan, Milan, Italy; ^3^ Department of Physiology, Paulista School of Medicine, Federal University of São Paulo (UNIFESP), São Paulo, Brazil

**Keywords:** autonomic nervous system, heart rate variability, hypoxemia, breathing, sex differences

## Abstract

**Introduction:**

Males and females differ anatomically and functionally in cardiorespiratory regulation, with males tending to experience greater oxygen desaturation under hypoxia. Therefore, sex might moderate cardiorespiratory responses to acute hypoxia exposure. Accordingly, we hypothesized that sex differences in cardiovascular and ventilatory responses would be more pronounced with equal hypoxia duration (iso-time) but less pronounced at similar oxygen desaturation levels (iso-saturation).

**Methods:**

Twenty-two (12 females) healthy individuals were exposed to normoxia (10 min at FiO_2_ = 0.21) and hypoxia (10 min at FiO_2_ = 0.115), respectively. Pulse oxygen saturation (SpO_2_), R-R intervals, cardiac output, blood pressure (BP), and ventilatory data were continuously recorded during spontaneous breathing. Spectral analysis of R-R intervals and systolic BP revealed cardiovascular autonomic modulation in the low- (LF; 0.04–0.15 Hz) and high-frequency (HF; 0.15–0.40 Hz) bands and alpha-index (α–LF) assessed spontaneous baroreflex sensitivity (BRS). Sex differences were compared in iso-saturation and iso-time analyses.

**Results:**

At 10 min of hypoxia (iso-time), males desaturated more than females (interaction: *p* = 0.004), and hypoxia-induced tachycardia in both groups (*p* < 0.001), but no “sex-time” interaction was found for cardiovascular data. In contrast, only males responded with ventilatory responses during iso-time hypoxia, decreasing breathing frequency (interaction: *p* = 0.022) and increasing tidal volume (Vt) (interaction: *p* = 0.036). Otherwise, during iso-saturation (SpO_2_-matched ∼91%), heart rate and LF of R-R intervals increased more in females than in males (interaction: *p* = 0.049). However, only males increased Vt (interaction; *p* = 0.037).

**Conclusion:**

Our data indicate that females counterbalance hypoxia mainly by systemic circulatory adjustments, while males use both, circulatory and ventilatory adjustments.

## 1 Introduction

High-altitude environments are well known to induce systemic hypoxia, a physiological stress that is not well tolerated by all humans (1–3). For a given inspired hypoxic level, individuals present a widely scattered oxygen saturation response (4, 5), and probably lower oxygen saturation contributes to hypoxic intolerance. A plausible factor related to inter-individual differences in the responses to hypoxia may be sex, as suggested by previous studies showing that females present more incidence of symptoms high altitude-related than males (6). Whether sex is a relevant factor in the inter-individual responsiveness to hypoxia, a further deep investigation into sex-related physiological differences to hypoxia is needed.

In normoxia, sex differences are notable in the lung's shape and size (7) and in the luminal area of large conducting airways, which are smaller in females than males (8). In the cardiovascular system, sex differences are present in anatomy, morphology, and regulation. For instance, compared to males, females have smaller hearts, higher heart rates, lower blood pressure (9), and greater peripheral vasodilation (10). However, in hypoxia, cardiorespiratory regulation remains controversial. Some studies have shown similar oxygen desaturation between sexes, with females presenting a higher heart rate and sympathetic activity (11). In contrast, although Boos et al. (12) also found a greater increase in heart rate in females than in males under hypoxia, oxygen saturation in females tended to be higher than in males. Higher oxygen saturation levels in females have been observed under hypoxia compared to males (13, 14). The reasons behind the controversial results involving sex differences in oxygen desaturation and cardiac responses remain unclear.

Overall, sex may be a relevant factor for the well-known inter-individual differences under hypoxia. The reasons for investigating sex differences are based on multiple anatomical and physiological aspects, especially under hypoxia since sex differences in chemoreflex have been widely discussed (15, 16). Also, inconclusive results on autonomic responses to hypoxia may stem from diverse methodological approaches, as exposure time and desaturation levels have not been considered confounding factors that could mask potential sex differences. Thus, we investigated whether sex differences in cardiorespiratory responses to hypoxia are due to distinct desaturation levels or different hypoxic responsiveness between males and females at the same desaturation level, focusing on iso-time and iso-saturation analyses to emphasize the roles of exposure time and desaturation in cardiorespiratory responses.

Considering that males tend to experience greater oxygen desaturation to short-term hypoxia (14), we tested the hypothesis that males would exhibit greater cardiovascular and ventilatory responses to hypoxia of equal duration (iso-time) due to greater desaturation compared to females. Additionally, at equal desaturation (iso-saturation), the sex differences in hypoxic responsiveness would be less pronounced but still present. Thus, we aimed to investigate the potential sex differences in cardiovascular autonomic modulation and ventilatory response at equivalent levels of oxygen desaturation (iso-saturation) and hypoxic exposure time (iso-time). This approach makes it possible to clarify if sex differences are related to oxygen desaturation or hypoxic responsiveness.

## 2 Methods

### 2.1 Participants

Twelve females and ten males, matched by age and BMI, participated as volunteers in this study (Table 1) after undergoing the anamnesis and electrocardiogram screening. The females who engaged in this study had not used contraceptives for at least 6 months. The group of females was assessed during the early follicular phase (i.e., the first week) of the menstrual cycle. The current study included healthy volunteers, lowlanders residents, and non-smokers who did not travel to high altitudes for at least 6-months previous to the present study. Individuals with any known cardiopulmonary or metabolic diseases were not included in the present investigation. The study was approved (number: 6.506.154) by the institutional ethics committee, registered in the public database Plataforma Brasil (CAAE: 28151519.0.0000.5243; National Council of Health, Ministry of Health, Brazil), and followed the Declaration of Helsinki. All participants signed the informed consent form.

**Table 1:** Participant's characteristics.

Variables	Males	Females	*t*-test
Age (years)	24 ± 3	25 ± 6	*p* = 0.64
height (m)	1.76 ± 0.07	1.64 ± 0.06	*p* < 0.001
weight (kg)	72.95 ± 10.31	59.96 ± 12.56	*p* = 0.02
BMI (kg.m^−2^)	23.5 ± 3.2	22.2 ± 3.7	*p* = 0.39
BS (m^2^)	1.89 ± 0.1	1.64 ± 0.1	*p* < 0.001

BMI, body mass index; BS, body surface area; mean ± SD.

### 2.2 Experimental protocol

**Figure 1:**
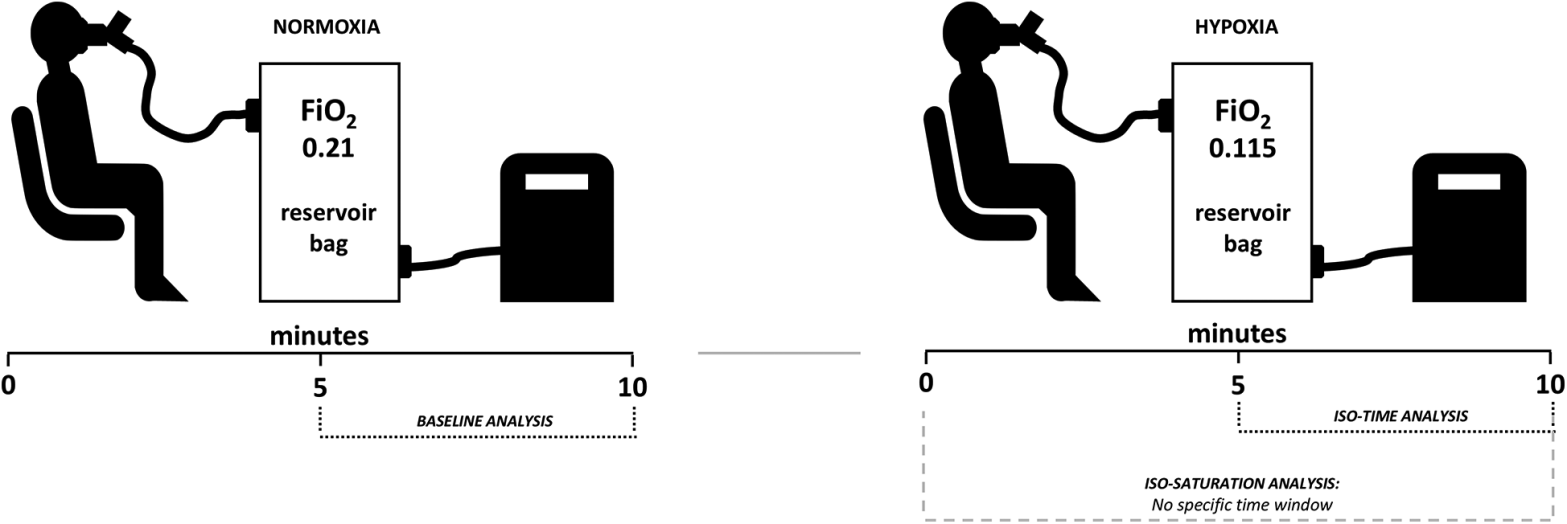
Illustration of the experimental design. As described in the methods, males and females underwent baseline normoxia followed by hypoxia, with both conditions lasting 10 min. Iso-time analysis was conducted at a fixed time, while iso-saturation analysis had no specific time window, aiming to analyze sexes at matched oxygen desaturation.

The experiments were conducted in the afternoon, between 2:00 and 4:00 p.m., in an air-conditioned laboratory room, with 50%–60% relative air humidity and temperatures between 20°C and 22°C (Kestrel 4500, Minneapolis, MN, USA). Upon arriving at the laboratory, participants rested for 20 min in a sitting position. During this time, beat–to–beat hemodynamic variables such as heart rate (HR), stroke volume (SV), cardiac output (CO), and blood pressure (BP) were recorded. Participants were also instrumented using a mask coupled to the spirometer flow head to record breathing frequency (BF), tidal volume (Vt), minute ventilation (VE), and a pulse oximeter for SpO_2_ monitoring. Thereafter, 10 min in NOR (FiO_2_ = 0.21 ∼sea level) and 10 min in HYP (FiO_2_ = 0.115 ∼4,800 m) (Figure 1).

### 2.3 Simulated altitude

The hypoxic condition was produced through an oxygen dilution system (Everest Summit II—Hypoxico®, NY, USA). Two hypoxic systems were coupled with two 200-L non-diffusing gas reservoirs, with one reservoir supplied with FiO_2_ of 0.21 (i.e., normoxia, sea-level equivalent) and the other with FiO_2_ of 0.115 (i.e., hypoxia∼4,800 m). The reservoirs contained an O_2_ monitor at their outputs to ensure accurate control of the FiO_2_ offered. In addition, the reservoirs were essential to ensure that the flow promoted by the hypoxic generators did not interfere with the ventilatory data recorded by the spirometer.

### 2.4 Experimental procedures

Heart rate, stroke volume, and cardiac output recordings were done through a noninvasive trans-thoracic bioimpedance device (17) (PhysioFlow®—Manatec Biomedical, FRA). The R-R intervals were recorded by an electrocardiogram signal (ECG Module®—FMS, NL), and the continuous blood pressure data were recorded by infrared photoplethysmography (Finometer® PRO—FMS, NL). The data acquisition frequency was sampled at 1 kHz. Minute ventilation (VE) was calculated as the product of breath-by-breath volume tidal (Vt; determined from the integral of the flow signal) and breathing frequency (BF; determined by the number of events per minute in the flow signal) recorded by the spirometer (Spirometer FE141—ADInstruments, NZL). All the devices were connected to a digital signal integrator (PowerLab®—ADInstruments, NZL) to allow the recordings would be synchronized through a specific software (LabChart 8.1 PRO—ADInstruments, NZL). A bidirectional valve (Y730—Hans Rudolph, USA) was connected at the outlet of the spirometer flow-head, allowing inhalation of gases with FiO_2_ of 0.21 or 0.115 from the reservoirs, and exhalation to room air. Before the start of the recordings, the spirometer was calibrated with a known volume (i.e., 3-L) through an appropriate syringe. Oxygen saturation data were assessed by a pulse oximeter (Oxi–Go® QuickCheck PRO—Oximeter Plus, USA).

### 2.5 Data analysis

For the heart rate variability (HRV) analysis, beat-to-beat time series of the R-R intervals were analyzed using a time window of 300 beats, focusing on the last 5 min for normoxia and iso-time hypoxia. In the case of iso-saturation hypoxia, the HRV segment was adjusted to match the period of equal desaturation. Spectral analysis was performed using Fast Fourier Transform (FFT) using Welch's method and a Hanning window (50% overlap), obtaining indexes in the frequency-domain as the total power (TP), the low-frequency component (LF, 0.04–0.15 Hz; often associated with sympathetic modulation), the high-frequency component (HF, 0.15–0.40 Hz; a marker of vagal modulation and synchronous with respiration), and the sympatho-vagal (LF/HF) balance. The LF and HF spectral powers were normalized by dividing the LF and HF values expressed in absolute units by the total power minus the VLF spectral power. Spontaneous cardiac baroreflex sensitivity (SBR) was analyzed by the alpha coefficient for the low component (α–LF) through the square root of the ratio of LF_R-R_ and LF from systolic blood pressure (LF_SBP_) powers (18). All HRV and BRS data were analyzed through *ad hoc* software (HeartScope II; A.M.P.S. LLC version 1.4.3.0., ITA).

Total vascular conductance (TVC) was calculated through the ratio between CO and mean blood pressure. Given the difference between males and females in body size, we normalized SV, CO and Vt by body surface area (BSA; in m^2^) = [0.20247 × height (in m) 0.725 × weight (in kg) 0.425] (19). Thus, stroke volume (SV), cardiac output (CO), and tidal volume (Vt) were normalized to body surface area, resulting in the following indexes: SVi, CI, and Vt/_BSA_, respectively.

### 2.6 Statistical analysis

The sample size was calculated for the following variables (iso-time: SpO_2_), and (iso-saturation: HR and Vt) considering a *β* of 0.80 and an *α* of 0.05, using an *ad hoc* software (G*Power 3.1.9.7; Heinrich-Heine-Universität, Düsseldorf, Germany). Data normality was tested using the Shapiro-Wilk test (see Supplementary Tables S1, S2). To compare the characteristics of the participants between males and females, an unpaired Student's *t*-test was carried out. The physiological changes provided by hypoxia were calculated by delta (Δ = hypoxia—normoxia) for both groups. The same test or the Mann-Whitney *U* test was used to compare the differences between deltas when appropriate. After that, the analysis of variance (ANOVA) for two factors (time and group) was done to evaluate hypoxia over time in both sexes. Iso-saturation and iso-time analyses were conducted separately using a mixed two-way ANOVA (group and condition), with repeated measures for the gas condition, to assess the main effects of hypoxia and sex differences and their interactions. Multiple comparisons were performed using the Bonferroni post-hoc test to find where the differences are. Graphics and statistical analysis were carried out through GraphPad (GraphPad Prism v 8.0.2, Inc., CA, USA). All data are presented as mean ± SD, and significance was set at *p* ≤ 0.05.

## 3 Results

The power analysis revealed large effect sizes (*f*) of 0.68 for SpO_2_, and 0.61 and 0.52 for HR and Vt, respectively, and that a sample size of 8–10 participants would be required for accurate data. Therefore, the sample size in this study meets the power analysis requirements. The results of normality tests may be seen in the supplementary data (Supplementary Tables S1, S2). The characteristic data of both groups were compared, showing that males and females were matched in age and body mass index, but, as expected, differed significantly in height, weight, and body surface (Table 1).

**Figure 2:**
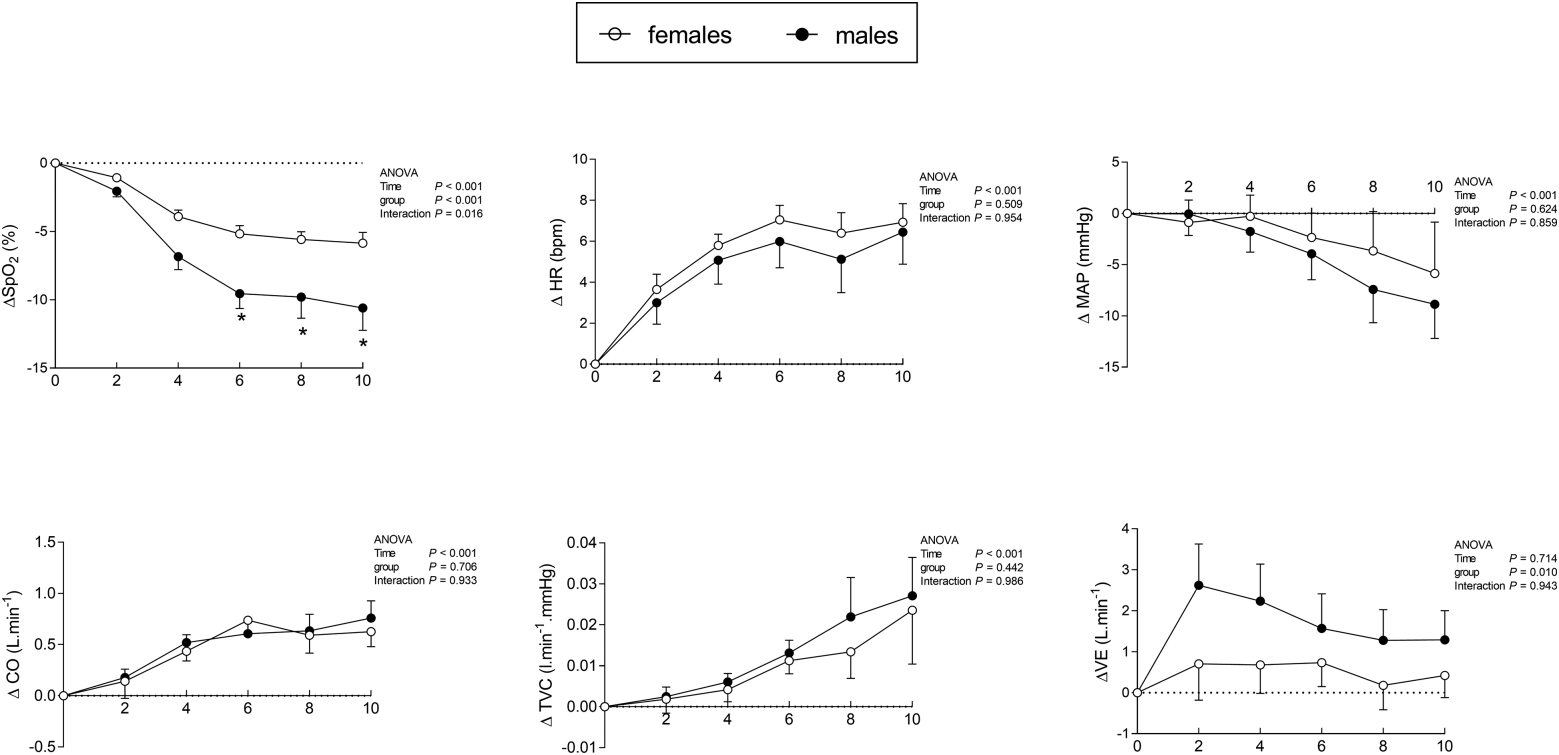
Cardiorespiratory responses in males and females averaged over each 2-minute interval during 10 min of hypoxia. Δ, difference (hypoxia—normoxia); ΔSpO_2_, oxygen desaturation; ΔHR, heart rate response; ΔMAP, mean arterial pressure; ΔCO, cardiac output; ΔTVC, total vascular conductance; ΔVE, minute ventilation. *p < 0.05 for interaction (time × group). Results are described as mean ± SE.

Regarding the time effect, an interaction between sex and time was observed only for oxygen desaturation (Figure 2). The ANOVA showed that the time effect of hypoxia was similar for both groups, increasing heart rate, cardiac output, and total vascular conductance while decreasing mean blood pressure. However, the ventilatory response was higher in males than in females, and was not influenced by time (Figure 2). Hemodynamic, autonomic, and ventilatory data for all participants are shown in Table 2, which presents sex-related comparisons for iso-saturation analysis, and in Table 3, which presents sex-related comparisons for iso-time hypoxia analysis.

**Table 2:** Cardiorespiratory responses in males and females during iso-saturation analysis.

Variables	Males		Females		*p* ANOVA		
	Normoxia	Iso-saturation (hypoxia)	Normoxia	Iso-saturation (hypoxia)	(Time)	(Sex)	(Int)
SpO_2__(%)_	98 ± 1	91 ± 3*	98 ± 1	92 ± 2*	0.000	0.237	0.125
Hemodynamic data							
HR_(bpm)_	74 ± 10	77 ± 8*	83 ± 6^#^	90 ± 7^†^^,^*^,^^#^	0.000	0.001	0.049
SV_(ml)_	89 ± 15	89 ± 14	83 ± 12	84 ± 13	0.531	0.363	0.788
SVi_(ml.m^2^__)_	47 ± 6	47 ± 5	51 ± 5	51 ± 5	0.587	0.119	0.782
CO_(L.min^−1^__)_	6.43 ± 0.76	6.85 ± 0.91	6.99 ± 0.96	7.46 ± 1.16*	0.002	0.207	0.641
CI_(L.min^−1^__.m^2^__)_	3.45 ± 0.33	3.64 ± 0.34	4.26 ± 0.42^#^	4.54 ± 0.55*^,^^#^	0.002	0.000	0.481
Autonomic data							
lnTP	8.9 ± 1.0	8.8 ± 0.9	7.8 ± 0.8^#^	7.4 ± 0.8^#^^,^*	0.042	0.002	0.085
LF_(n.u)_	29.7 ± 16.5	32.6 ± 17.0	24.7 ± 11.6	37.7 ± 18.1^†^^,^*	0.005	0.996	0.049
HF_(n.u)_	64.0 ± 15.7	57.6 ± 13.9	66.9 ± 14.6	53.2 ± 18.4*	0.002	0.911	0.210
LF/HF	0.6 ± 0.5	0.7 ± 0.7	0.4 ± 0.4	0.9 ± 0.8*	0.021	0.797	0.176
LF_SBP(mmHg^2^__)_	24.8 ± 23.6	33.5 ± 37.9	5.4 ± 2.3	12.3 ± 11.2	0.018	0.043	0.768
α–LF_(ms.mmHg^−1^__)_	10.1 ± 5.6	9.0 ± 5.3	10.0 ± 4.0	6.7 ± 2.5*	0.001	0.524	0.058
Ventilatory data							
BF_(cycles.min^−1^__)_	15 ± 3	15 ± 3	17 ± 3	17 ± 3	0.724	0.162	0.099
V_t__(ml)_	999 ± 180	1,120 ± 206^†^^,^*	747 ± 125^#^	753 ± 116^#^	0.023	0.000	0.037
VE_(L.min^−1^__)_	15.16 ± 2.47	16.72 ± 3.64*	12.44 ± 2.69	12.87 ± 2.56^#^	0.035	0.012	0.213

Values described in mean ± SD. SpO_2_, pulse oxygen saturation; HR, heart rate; SV, stroke volume; SVi, stroke volume index is the SV calculated by the ratio to body surface area; CO, cardiac output; CI, cardiac index is the CO calculated by the ratio to body surface area; lnTP, total power; LF, low frequency component of R-R intervals; HF, high frequency component of R-R intervals; LF/HF, sympathovagal balance; LF_SBP_, low frequency component of blood pressure; α–LF, alpha index of low-frequency components [i.e., √ LF_R-R_/LF_SBP_]; BF, breathing frequency; Vt, tidal volume; VE, minute ventilation. Bonferroni post-hoc analysis, time (normoxia vs. iso-saturation hypoxia) *p ≤ 0.05; sex = males vs. females, ^#^*p* ≤ 0.05; int = time-group interaction ^†^*p* ≤ 0.05.

**Table 3:** Cardiorespiratory responses in males and females during iso-time analysis.

Variables	Males		Females		*p* ANOVA		
	Normoxia	Iso-time (hypoxia)	Normoxia	Iso-time (hypoxia)	(Time)	(Sex)	(Int)
SpO_2__(%)_	98 ± 1	87 ± 5^†^^,^*^,^^#^	98 ± 1	92 ± 2*	0.000	0.007	0.004
Hemodynamic data							
HR_(bpm)_	74 ± 10	80 ± 8*	83 ± 6^#^	90 ± 7*^,^^#^	0.000	0.003	0.282
SV_(ml)_	89 ± 15	89 ± 14	83 ± 12	84 ± 13	0.519	0.360	0.798
SVi_(ml.m^2^__)_	47 ± 6	47 ± 5	51 ± 5	51 ± 5	0.586	0.118	0.781
CO_(L.min^−1^__)_	6.43 ± 0.76	7.02 ± 0.96*	6.99 ± 0.96	7.46 ± 1.16*	0.000	0.390	0.388
CI_(L.min^−1^__.m^2^__)_	3.45 ± 0.33	3.82 ± 0.46*	4.26 ± 0.42^#^	4.54 ± 0.55*^,^^#^	0.000	0.000	0.562
Autonomic data							
lnTP	8.9 ± 1.0	8.7 ± 0.9	7.8 ± 0.8^#^	7.4 ± 0.8^#^^,^*	0.021	0.003	0.185
LF_(n.u)_	29.7 ± 16.5	38.1 ± 18.3	24.7 ± 11.6	37.7 ± 18.1*	0.002	0.666	0.438
HF_(n.u)_	64.0 ± 15.7	54.3 ± 15.3	66.9 ± 14.6	53.2 ± 18.4*	0.001	0.886	0.497
LF/HF	0.6 ± 0.5	0.9 ± 0.7	0.4 ± 0.4	0.9 ± 0.8*	0.007	0.906	0.495
LF_SBP(mmHg^2^__)_	24.8 ± 23.6	46.5 ± 40.3*	5.4 ± 2.3	12.3 ± 11.2^#^	0.002	0.011	0.075
α–LF_(ms.mmHg^−1^__)_	10.1 ± 5.6	8.1 ± 5.6	10.0 ± 4.0	6.7 ± 2.5*	0.000	0.693	0.288
Ventilatory data							
BF_(cycles.min^−1^__)_	15 ± 3	14 ± 2^†^	17 ± 3	17 ± 3	0.650	0.089	0.022
V_t__(ml)_	999 ± 180	1,125 ± 197^†^^,^*	747 ± 125^#^	753 ± 116^#^	0.023	0.000	0.036
VE_(L.min^−1^__)_	15.16 ± 2.47	16.40 ± 3.21	12.44 ± 2.69	12.87 ± 2.56^#^	0.055	0.012	0.335

Values described in mean ± SD. SpO_2_, pulse oxygen saturation; HR, heart rate; SV, stroke volume; SVi, stroke volume index; CO, cardiac output; CI, cardiac index; lnTP, total power; LF, low frequency component of R-R intervals; HF, high frequency component of R-R intervals; LF/HF, sympathovagal balance; LF_SBP_, low frequency component of blood pressure; α–LF, alpha index of low-frequency components [i.e., √ LF_R-R_/LF_SBP_]; BF, breathing frequency; Vt, tidal volume; VE, minute ventilation. Bonferroni post-hoc analysis, time (normoxia vs. iso-time hypoxia) *p ≤ 0.05; sex = males vs. females, ^#^*p* ≤ 0.05; int = time-group interaction ^†^*p* ≤ 0.05.

Iso-saturation period analysis (i.e., matched SpO_2_; see Table 2) was confirmed by the absence of differences in SpO_2_ levels between males and females under hypoxia in this analysis segment. Herein, females showed an increased heart rate and low-frequency component of R-R intervals and a trend to reduce baroreflex sensitivity (α–LF) during iso-saturation hypoxia compared to males. On the other hand, ventilatory data remained unchanged in females, while only males increased tidal volume under iso-saturation, as evidenced by the sex-condition interaction. The other physiological data presented only the main effects of ANOVA, without interactions, such as those related to hypoxia showed increases in heart rate, cardiac output, and low-frequency bands of R-R intervals and blood pressure, as well as a decrease in baroreflex sensitivity, total power, and the high-frequency band of R-R intervals, and increases in tidal volume and ventilation in response to iso-saturation hypoxia (Table 2). Additionally, other main effects such as those related to sex as a factor, showed that females had a higher heart rate and cardiac output index but lower tidal volume, ventilation, and low-frequency band of blood pressure (Table 2). Sex differences are also found for the relative tidal volume (i.e., indexed by body surface area) in response to hypoxia when comparing this variable between sexes by delta (ΔVt/_BSA_; males: 0.062 ± 0.077 vs. females: 0.007 ± 0.053 L/m^2^; *p* = 0.03).

During iso-time analysis (i.e., matched timeframes; see Table 3), an interaction effect was observed for oxygen saturation, showing that males desaturated more than females. Other interactions from ANOVA revealed that only males increased tidal volume and decreased breathing frequency. Further main effects related to iso-time hypoxia included increases in heart rate, cardiac output, the low-frequency band of R-R intervals and blood pressure, and vascular conductance, as well as decreases in the high-frequency band of R-R intervals and baroreflex sensitivity. Sex-related main effects from the ANOVA revealed that females had higher heart rates, cardiac output, and total vascular conductance, but lower tidal volume, ventilation, and the low-frequency band of blood pressure (Table 3). In addition, males showed a greater response of relative tidal volume to iso-time hypoxia than females, as shown by delta (ΔVt/_BSA_; males: 0.064 ± 0.082 vs. females: 0.007 ± 0.053 L/m^2^; *p* = 0.03).

## 4 Discussion

The present study investigated sex differences in cardiorespiratory responses to hypoxia, focusing on oxygen saturation levels and time under hypoxia. The main findings in this investigation were: (1) under hypoxic iso-saturation, females had higher heart rates and greater low-frequency band response of HRV compared to males. Whereas, males presented a higher tidal volume in response to hypoxic iso-saturation than females. (2) The iso-time hypoxic analyses revealed that females defended oxygen saturation over time better compared to males. Despite different levels of desaturation, there were no sex differences in cardiovascular responses to iso-time hypoxia, but males needed ventilatory compensation by presenting greater tidal volume under iso-time hypoxia, whereas females did not. Collectively, these findings suggest that in short-term normobaric hypoxia, females preserve oxygen saturation and counterbalance hypoxemia mainly through tachycardia. Differently, males achieve lower saturation levels and even tachycardia and higher tidal volume fail to maintain preserved oxygen saturation. These results provide evidence that males and females exhibit distinct modulation of cardiorespiratory responses to acute hypoxia.

### 4.1 Sex differences and oxygen desaturation

Oxygen desaturation diverges between sexes over time, as shown by some studies (12–14, 20), although this finding is not reported in all studies (21–23). Boos and colleagues (12) in a study involving 14 individuals (seven females), found that females tend to remain with higher oxygen saturation than males after 150 min of normobaric hypoxia. It is well known that pulmonary vessels when exposed to hypoxia can lead to blood flow diversion from hypoxic to non-hypoxic lung areas (i.e., hypoxic pulmonary vasoconstriction) preventing at least in part the arterial deoxygenation (24). Despite Boos and colleagues (12) having shown that pulmonary vascular resistance was increased by hypoxia, the effect occurred similarly in both sexes (12). In our study, pulmonary circulation was not evaluated, but in females, the absence of ventilatory responses to hypoxia (Tables 2, 3) might have provided a lower rate of alveolar gas turnover between the hypoxic gas from the mask to the alveoli. Concomitantly, even with a slight desaturation (Figure 2), females presented tachycardia, increasing cardiac output (Figure 2), which perhaps may contribute to an augment in blood flow velocity in alveolar-capillary units leading to a lower time to hematosis. Importantly, in normoxia, adult females may also present higher oxygen saturation than males, which is not evident in newborns (25) and prepubertal children (26), remaining unknown the contribution of female hormones in oxyhemoglobin affinity. However, sex differences in oxygen saturation in normoxia were not found in the present study.

In humans, previous studies have shown an inverse relation between hypoxic ventilatory response and hypoxic pulmonary vasoconstriction (27), which means that less ventilatory response, more pulmonary vasoconstriction under hypoxia. In a certain way, this mechanism could offer a short-term protection to desaturation, but if this condition continues for more time, it could lead to pulmonary hypertension, right heart failure, and edema. However, the defense of oxygen saturation in females might be related to other mechanisms, because in contrast, female hormones, such as estrogen, seem to contribute to pulmonary vasodilation, as shown in animal models, attenuating hypoxic pulmonary vasocontriction (24). Another possibility would be the oxygen affinity to hemoglobin, however, previous studies showed that females have lower oxygen affinity to hemoglobin than males, which is attributed to higher 2,3-diphosphoglycerate levels (28). Nevertheless, blood gases and hematocrit were not measured in the current study, which limits our interpretation. Therefore, the mechanisms behind a higher saturation under hypoxia in females deserve further investigation.

### 4.2 Sex-related differences in cardiac autonomic control in normoxia

Our results revealed that females had higher heart rates and lower total R-R variability compared to males under baseline normoxic conditions (Table 2), partially corroborating with the literature that typically reports the same result in females, but paradoxically also reports a relative vagal dominance in females, while in males reports lower heart rates with a relative sympathetic dominance (29). In our data, no sex differences were found for sympatho-vagal balance in normoxia, despite females having a lower baseline mean of LF/HF (Table 2). Additionally, in normoxia, females had lower sympathetic vascular modulation (i.e., estimated by LF spectral component of systolic blood pressure), but similar spontaneous cardiac baroreflex sensitivity compared to males (Table 2). An in-depth discussion of sex-specific cardiovascular autonomic modulation is beyond the scope of this investigation. Briefly, these sex differences may be partially attributed to the effects of estrogen, oxytocin-stimulated neurons affecting vagal tone, amygdala activity, sex-related differences in brain structures (29), and the predominance of beta-adrenergic vasodilation in young females (30). Notably, autonomic activity may be influenced by several other factors, and in the context of biological sex differences, intra-individual variability should be considered, particularly in females due to potential differences in autonomic activity across different phases of the menstrual cycle (16).

### 4.3 Sex-related differences in cardiac autonomic control in hypoxia

During iso-time analysis, our results demonstrated increased heart rate in both, males and females under hypoxia, characterized by vagal withdrawal and augmented sympathetic modulation, shifting the sympatho-vagal balance towards sympathetic dominance (Table 3). Additionally, we observed a reduction in spontaneous baroreflex sensitivity in both sexes under iso-time hypoxia. Importantly, these cardiovascular autonomic responses were consistent in females, but were only observed in males when they exhibited lower oxygen saturation (Table 3). Previous findings indicate that the augmented heart rate under hypoxia is often a result of vagal withdrawal, as triggered by the aortic bodies (31, 32). Cardiac vagal withdrawal under hypoxia was also reported by other investigations (4, 21, 33), and when sex was compared no differences were noted in cardiac vagal modulation (21).

During iso-saturation (SpO_2_-matched) analysis, we found that females exhibited higher heart rates, increased cardiac sympathetic modulation, and a tendency towards lower spontaneous baroreflex sensitivity (Table 2). Our findings contrast with those of Botek and colleagues (21), who showed that males exhibited relatively higher cardiac sympathetic responses compared to females under short-term severe hypoxia with similar oxygen desaturation levels. The hypoxic level used by Botek et al. (21) was considerably more severe (FiO_2_ = 0.096) than in the current investigation (FiO_2_ = 0.115). Overall, the average oxygen saturation achieved by males and females in Botek et al.'s study (21) was near 70%, with considerable dispersion, whereas in the current study, iso-saturation analysis was near SpO_2_ 91%, and in the iso-time analysis, only males reached an average saturation near 87%, with more discrete dispersion. These differences are important because a stronger hypoxic stimulus contributes to greater oxygen desaturation dispersion among participants but might overshadow potential differences in cardiovascular autonomic sensitivity that could be observed under moderate levels of hypoxemia.

It is known that hypoxia increases sympathoexcitation, but the results are inconclusive when sex differences are compared. A study that measured peripheral sympathetic activity directly showed that females may exhibit faster sympathoexcitation (11), while another study found that the sympathetic response of females to isocapnic hypoxia is similar to that of males (15). These studies measured peripheral sympathetic activity directly, so it is not appropriate to compare their results directly with ours, even though the normalized low-frequency band of the R-R intervals has been correlated with sympathetic activity (34, 35). Herein, although the normalized LF of the R-R interval increased more in females during iso-saturation hypoxia, suggesting a possible greater sympathetic predominance over heart rate compared to males, this phenomenon needs to be further investigated using a similar protocol that integrates HRV markers and direct sympathetic measurements to explore the underlying mechanisms in sex differences in cardiovascular rhythms to hypoxia.

### 4.4 Sex differences and ventilatory response

In contrast with males, females did not change ventilation in response to hypoxia in both segment analyses (iso-saturation and iso-time), which was intriguing. The absence of ventilatory response to hypoxia in females contrasts with previous studies (14), and it likely may not be explained by higher oxygen saturation levels because males increased the tidal volume in SpO_2_-matched analysis. In a longer exposure to moderate normobaric hypoxia for 7 h, Camacho-Cardenosa and co-authors (36) showed a robust increase in ventilation in males and females, however, comparing the sexes, it was significantly greater in males. In our study, ventilation did not differ between sexes, but males tend to present greater averages. Conversely, tidal volume markedly increased in response to short-term hypoxia in males. Additionally, only males reduced breathing frequency in iso-time hypoxia (Table 3), which supports previous investigations in humans suggesting that sex influences breathing frequency response to hypoxia (14). The mechanisms underlying sex differences in hypoxic ventilatory response in humans remain largely unknown. Previous studies suggest that increased circulating female hormones play a role in an enhanced hypoxic ventilatory response (37, 38). However, in our study, females were assessed during the low-hormone phase (i.e., the early follicular phase) of the menstrual cycle, thereby reducing the potential effect on ventilatory response. Sex hormones appear to act directly on the peripheral chemoreflex, as experiments in rats have shown that ovarian steroids can influence breathing by decreasing dopaminergic inhibitory activity in the carotid bodies (39). However, the differences in hypoxic response may extend beyond sex, as there are species variations in the mechanisms underlying the peripheral chemoreflex (40), which makes studying this in humans essential.

A matter of future debate should address additional factors contributing to inter- and intra-individual differences in physiological responses to hypoxia. These factors include a detailed investigation of anatomical and functional differences between sexes, the influence of different phases of the menstrual cycle, and follow-up studies to assess the impact of aging and various clinical populations.

### 4.5 Limitations

This study has some limitations that should be acknowledged. First, females were evaluated during the follicular phase of the menstrual cycle, but this was self-reported based on their cycle calendars. Therefore, estrogen and progesterone levels were not measured in this investigation. Second, health status was assessed through anamnesis and electrocardiogram, with participants self-reporting as healthy. However, the participants did not show any signs of cardiorespiratory alterations that could be considered abnormal before or during the tests. Third, the lack of end-tidal gas recordings (i.e., PETO_2_ and PETCO_2_) and the absence of carbon dioxide supplementation to maintain isocapnia represent significant limitations. However, the model of hypoxia used here (i.e., poikilocapnic hypoxia, or no CO_2_ addition) has shown results consistent with isocapnic hypoxia with regard to cardiovascular responsiveness (41, 42). Additionally, the hypoxic condition in this study has higher external validity, as in natural hypoxic environments, carbon dioxide is not artificially added to the hypoxic gas to maintain isocapnia. Fourth, venous and arterial blood samples were not collected, limiting interpretations as hematocrit and arterial gases (PaO_2_, PaCO_2_) would provide valuable data. However, for our objectives, only oxygen saturation offers information in real time, making it possible to select the correct time window analysis for iso-time and iso-hypoxemia. Lastly, the current findings on sex differences should not be extrapolated to clinical contexts, taking into account that further investigations involving specific diseases, considering factors beyond sex such as age, obesity, and comorbidities, are needed.

## 5 Conclusion

In conclusion, our data indicate that females primarily counterbalance hypoxia through systemic circulatory adjustments, preserving oxygen saturation, while males rely on both circulatory and ventilatory adjustments but experience greater oxygen desaturation. Therefore, sex differences should be considered a significant factor that may considerably influence cardiorespiratory adjustments during hypoxia.

## Data Availability

The original contributions presented in the study are included in the article/Supplementary Material, further inquiries can be directed to the corresponding author.

## References

[1] Karinen HM Peltonen JE Kähönen M Tikkanen HO. Prediction of acute mountain sickness by monitoring arterial oxygen saturation during ascent. High Alt Med Biol. (2010) 11(4):325–32. 10.1089/ham.2009.1060 21190501

[2] Karinen HM Uusitalo A Vähä-Ypyä H Kähönen M Peltonen JE Stein PK Heart rate variability changes at 2400 m altitude predicts acute mountain sickness on further ascent at 3000-4300 m altitudes. Front Physiol. (2012) 3:336. 10.3389/fphys.2012.00336 22969727 PMC3431006

[3] Mairer K Wille M Grander W Burtscher M. Effects of exercise and hypoxia on heart rate variability and acute mountain sickness. Int J Sports Med. (2013) 34(8):700–6. 10.1055/s-0032-1327577 23386424

[4] Botek M Krejčí J De Smet S Gába A McKune AJ. Heart rate variability and arterial oxygen saturation response during extreme normobaric hypoxia. Auton Neurosci. (2015) 190:40–5. 10.1016/j.autneu.2015.04.001 25907329

[5] Krejčí J Botek M McKune AJ. Dynamics of the heart rate variability and oxygen saturation response to acute normobaric hypoxia within the first 10 min of exposure. Clin Physiol Funct Imaging. (2018) 38(1):56–62. 10.1111/cpf.12381 27380961

[6] MacInnis MJ Carter EA Freeman MG Pandit BP Siwakoti A Subedi A A prospective epidemiological study of acute mountain sickness in Nepalese pilgrims ascending to high altitude (4380 m). PLoS One. (2013) 8(10):e75644. 10.1371/journal.pone.0075644 24130729 PMC3794000

[7] Torres-Tamayo N García-Martínez D Lois Zlolniski S Torres-Sánchez I García-Río F Bastir M. 3D analysis of sexual dimorphism in size, shape and breathing kinematics of human lungs. J Anat. (2018) 232(2):227–37. 10.1111/joa.12743 29148039 PMC5770305

[8] Dominelli PB Ripoll JG Cross TJ Baker SE Wiggins CC Welch BT Sex differences in large conducting airway anatomy. J Appl Physiol. (2018) 125(3):960–5. 10.1152/japplphysiol.00440.2018 30024341 PMC6335094

[9] Prabhavathi K Selvi KT Poornima KN Sarvanan A. Role of biological sex in normal cardiac function and in its disease outcome - a review. J Clin Diagn Res. (2014) 8(8):BE01–4. 10.7860/JCDR/2014/9635.4771 25302188 PMC4190707

[10] Barnes JN. Sex-specific factors regulating pressure and flow. Exp Physiol. (2017) 102(11):1385–92. 10.1113/EP086531 28799254 PMC5665704

[11] Jones PP Davy KP Seals DR. Influence of gender on the sympathetic neural adjustments to alterations in systemic oxygen levels in humans. Clin Physiol. (1999) 19(2):153–60. 10.1046/j.1365-2281.1999.00158.x 10200897

[12] Boos CJ Mellor A O'Hara JP Tsakirides C Woods DR. The effects of sex on cardiopulmonary responses to acute normobaric hypoxia. High Alt Med Biol. (2016) 17(2):108–15. 10.1089/ham.2015.0114 27008376

[13] Riveros-Rivera A Penzel T Gunga HC Opatz O Paul F Klug L Hypoxia differentially affects healthy men and women during a daytime nap with a dose-response relationship: a randomized, cross-over pilot study. Front Physiol. (2022) 13:899636. 10.3389/fphys.2022.899636 35685284 PMC9171024

[14] Rispen L Marks D Green S. Dynamic ventilatory responses of females and males to acute isocapnic and poikilocapnic hypoxia. Respir Physiol Neurobiol. (2017) 245:57–64. 10.1016/j.resp.2017.05.005 28552789

[15] Sayegh ALC Fan JL Vianna LC Dawes M Paton JFR Fisher JP. Sex differences in the sympathetic neurocirculatory responses to chemoreflex activation. J Physiol. (2022) 600(11):2669–89. 10.1113/JP282327 35482235 PMC9324851

[16] Usselman CW Gimon TI Nielson CA Luchyshyn TA Coverdale NS Van Uum SH Menstrual cycle and sex effects on sympathetic responses to acute chemoreflex stress. Am J Physiol Heart Circ Physiol. (2015) 308(6):H664–71. 10.1152/ajpheart.00345.2014 25527774 PMC4360053

[17] Siebenmann C Rasmussen P Sørensen H Zaar M Hvidtfeldt M Pichon A Cardiac output during exercise: a comparison of four methods. Scand J Med Sci Sports. (2015) 25(1):e20–7. 10.1111/sms.12201 24646113

[18] Parati G Di Rienzo M Mancia G. How to measure baroreflex sensitivity: from the cardiovascular laboratory to daily life. J Hypertens. (2000) 18(1):7–19. 10.1097/00004872-200018010-00003 10678538

[19] Du Bois D Du Bois EF. A formula to estimate the approximate surface area if height and weight be known. 1916. Nutrition. (1989) 5(5):303–11; discussion 312–3. 2520314

[20] Nishimura T Ugarte J Ohnishi M Nishihara M Alvarez G Yasukochi Y Individual variations and sex differences in hemodynamics with percutaneous arterial oxygen saturation (SpO2) in young Andean highlanders in Bolivia. J Physiol Anthropol. (2020) 39(1):31. 10.1186/s40101-020-00240-y 33028423 PMC7542971

[21] Botek M Krejčí J McKune A. Sex differences in autonomic cardiac control and oxygen saturation response to short-term normobaric hypoxia and following recovery: effect of aerobic fitness. Front Endocrinol. (2018) 9:697. 10.3389/fendo.2018.00697 PMC6265316 30532736

[22] Nishimura T Arima H Koirala S Ito H Yamamoto T. Individual variations and sex differences in hemodynamics and percutaneous arterial oxygen saturation (SpO2) in Tibetan highlanders of Tsarang in the Mustang district of Nepal. J Physiol Anthropol. (2022) 41(1):9. 10.1186/s40101-022-00282-4 35292118 PMC8925233

[23] Miller AJ Cui J Luck JC Sinoway LI Muller MD. Age and sex differences in sympathetic and hemodynamic responses to hypoxia and cold pressor test. Physiol Rep. (2019) 7(2):e13988. 10.14814/phy2.13988 30659773 PMC6339536

[24] Sylvester JT Shimoda LA Aaronson PI Ward JPT. Hypoxic pulmonary vasoconstriction. Physiol Rev. (2012) 92:367–520. 10.1152/physrev.00041.2010 22298659 PMC9469196

[25] Levental S Picard E Mimouni F Joseph L Samuel TY Bromiker R Sex-linked difference in blood oxygen saturation. Clin Respir J. (2018) 12(5):1900–4. 10.1111/crj.12753 29227023

[26] Frimer Z Goldberg S Joseph L Picard E. Are there gender differences in blood oxygen saturation in prepubertal children? Clin Respir J. (2021) 15(6):657–60. 10.1111/crj.13340 33590698

[27] Albert TJ Swenson ER. Peripheral chemoreceptor responsiveness and hypoxic pulmonary vasoconstriction in humans. High Alt Med Biol. (2014) 15(1):15–20. 10.1089/ham.2013.1072 24444139

[28] Balcerek B Steinach M Lichti J Maggioni MA Becker PN Labes R A broad diversity in oxygen affinity to haemoglobin. Sci Rep. (2020) 10(1):16920. 10.1038/s41598-020-73560-9 33037242 PMC7547706

[29] Koenig J Thayer JF. Sex differences in healthy human heart rate variability: a meta-analysis. Neurosci Biobehav Rev. (2016) 64:288–310. 10.1016/j.neubiorev.2016.03.007 26964804

[30] Joyner MJ Wallin BG Charkoudian N. Sex differences and blood pressure regulation in humans. Exp Physiol. (2016) 101(3):349–55. 10.1113/EP085146 26152788

[31] Tubek S Niewinski P Reczuch K Janczak D Rucinski A Paleczny B Effects of selective carotid body stimulation with adenosine in conscious humans. J Physiol. (2016) 594:6225–40. 10.1113/JP272109 27435894 PMC5088231

[32] Siebenmann C Ryrsø CK Oberholzer L Fisher JP Hilsted LM Rasmussen P Hypoxia-induced vagal withdrawal is independent of the hypoxic ventilatory response in men. J Appl Physiol. (2019) 126(1):124–31. 10.1152/japplphysiol.00701.2018 30496709

[33] Buchheit M Richard R Doutreleau S Lonsdorfer-Wolf E Brandenberger G Simon C. Effect of acute hypoxia on heart rate variability at rest and during exercise. Int J Sports Med. (2004) 25(4):264–9. 10.1055/s-2004-819938 15162245

[34] DeBeck LD Petersen SR Jones KE Stickland MK. Heart rate variability and muscle sympathetic nerve activity response to acute stress: the effect of breathing. Am J Physiol Regul Integr Comp Physiol. (2010) 299(1):R80–91. 10.1152/ajpregu.00246.2009 20410469 PMC5017870

[35] Pagani M Montano N Porta A Malliani A Abboud FM Birkett C Relationship between spectral components of cardiovascular variabilities and direct measures of muscle sympathetic nerve activity in humans. Circulation. (1997) 95(6):1441–8. 10.1161/01.CIR.95.6.1441 9118511

[36] Camacho-Cardenosa A Camacho-Cardenosa M Tomas-Carus P Timón R Olcina G Burtscher M. Acute physiological response to a normobaric hypoxic exposure: sex differences. Int J Biometeorol. (2022) 66(7):1495–504. 10.1007/s00484-022-02298-y 35585281

[37] Moore LG McCullough RE Weil JV. Increased HVR in pregnancy: relationship to hormonal and metabolic changes. J Appl Physiol. (1987) 62:158–63. 10.1152/jappl.1987.62.1.158 3104285

[38] Regensteiner JG Woodard WD Hagerman DD Weil JV Pickett CK Bender PR Combined effects of female hormones and metabolic rate on ventilatory drives in women. J Appl Physiol. (1989) 66(2):808–13. 10.1152/jappl.1989.66.2.808 2540141

[39] Joseph V Soliz J Soria R Pequignot J Favier R Spielvogel H Dopaminergic metabolism in carotid bodies and high-altitude acclimatization in female rats. Am J Physiol Regul Integr Comp Physiol. (2002) 282(3):R765–73. 10.1152/ajpregu.00398.2001 11832397

[40] Zera T Moraes DJA da Silva MP Fisher JP Paton JFR. The logic of carotid body connectivity to the brain. Physiology. (2019) 34(4):264–82. 10.1152/physiol.00057.2018 31165684

[41] Halliwill JR Minson CT. Effect of hypoxia on arterial baroreflex control of heart rate and muscle sympathetic nerve activity in humans. J Appl Physiol. (2002) 93(3):857–64. 10.1152/japplphysiol.01103.2001 12183478

[42] Halliwill JR Morgan BJ Charkoudian N. Peripheral chemoreflex and baroreflex interactions in cardiovascular regulation in humans. J Physiol. (2003) 552(Pt 1):295–302. 10.1113/jphysiol.2003.050708 12897165 PMC2343329

