# Supplementary material for: Sex differences in cardiorespiratory control under hypoxia: the roles of oxygen desaturation and hypoxic exposure time
Source: Front Cardiovasc Med. 2025 Jan 31;12:1473910. doi: 10.3389/fcvm.2025.1473910 (PMC11825819; doi:10.3389/fcvm.2025.1473910)
Supplement: Supplementary file 1 [file Table1.docx]

Supplementary Material

**Table S1:** Shapiro-Wilk Test (p-values results).

|  | Males | | Females | |
| --- | --- | --- | --- | --- |
| Variable | **normoxia** | **hypoxia** | **normoxia** | **hypoxia** |
| SpO_2_ | 0.126 | 0.784 | 0.454 | 0.593 |
| HR | 0.464 | 0.361 | 0.164 | 0.526 |
| SV | 0.681 | 0.400 | 0.806 | 0.863 |
| CO | 0.402 | 0.502 | 0.911 | 0.974 |
| LF | 0.392 | 0.891 | 0.933 | 0.745 |
| HF | 0.835 | 0.991 | 0.184 | 0.496 |
| LF/HF | 0.035 | 0.045 | 0.008 | 0.062 |
| α-LF | 0.083 | 0.002 | 0.323 | 0.160 |
| BF | 0.508 | 0.205 | 0.239 | 0.124 |
| Vt | 0.063 | 0.210 | 0.131 | 0.271 |
| VE | 0.762 | 0.132 | 0.145 | 0.201 |

The results of the normality tests for normoxia and hypoxia. When the '*p*-value' is higher than 0.05, the data is normally distributed. Most of the data were normally distributed.

**Table S2:** Shapiro-Wilk test p-values for deltas in **Figure 2**:

|  | Males (p-values) | Females (p-values) |
| --- | --- | --- |
| ∆SpO_2_ | 0.185 | 0.170 |
| ∆HR | 0.193 | 0.075 |
| ∆MAP | 0.311 | 0.439 |
| ∆CO | 0.346 | 0.457 |
| ∆TVC | 0.539 | 0.525 |
| ∆VE | 0.694 | 0.222 |

Results of normality tests for deltas in response to hypoxia over time in males and females. The data were normally distributed as the 'p-values' of Shapiro-Wilk's test were higher than 0.05.
